# Aquaporins and Brain Tumors

**DOI:** 10.3390/ijms17071029

**Published:** 2016-06-29

**Authors:** Rosario Maugeri, Gabriella Schiera, Carlo Maria Di Liegro, Anna Fricano, Domenico Gerardo Iacopino, Italia Di Liegro

**Affiliations:** 1 Department of Experimental Biomedicine and Clinical Neurosciences (BIONEC), University of Palermo, Palermo I-90127, Italy; rosario.maugeri1977@gmail.com (R.M.); anna.fricano@unipa.it (A.F.); gerardo.iacopino@unipa.it (D.G.I.); 2 Department of Biological Chemical and Pharmaceutical Sciences and Technologies (STEBICEF), University of Palermo (UNIPA), Palermo I-90128, Italy; gabriella.schiera@unipa.it (G.S.); carlomaria.diliegro@unipa.it (C.M.D.L.)

**Keywords:** aquaporins (AQPs), brain tumors, glioblastoma multiforme, blood–brain barrier (BBB), extracellular vesicles (EVs)

## Abstract

Brain primary tumors are among the most diverse and complex human cancers, and they are normally classified on the basis of the cell-type and/or the grade of malignancy (the most malignant being glioblastoma multiforme (GBM), grade IV). Glioma cells are able to migrate throughout the brain and to stimulate angiogenesis, by inducing brain capillary endothelial cell proliferation. This in turn causes loss of tight junctions and fragility of the blood–brain barrier, which becomes leaky. As a consequence, the most serious clinical complication of glioblastoma is the vasogenic brain edema. Both glioma cell migration and edema have been correlated with modification of the expression/localization of different isoforms of aquaporins (AQPs), a family of water channels, some of which are also involved in the transport of other small molecules, such as glycerol and urea. In this review, we discuss relationships among expression/localization of AQPs and brain tumors/edema, also focusing on the possible role of these molecules as both diagnostic biomarkers of cancer progression, and therapeutic targets. Finally, we will discuss the possibility that AQPs, together with other cancer promoting factors, can be exchanged among brain cells via extracellular vesicles (EVs).

## 1. Introduction

Central Nervous System (CNS) tumors are among the most complex human cancers and constitute a group of anatomically similar, but still diverse tumor species with different morphology, etiology, site of origin, molecular biology, and clinical progression [1].

Most primary cancers of the adult brain originate from glial cells or glial cell precursors and are called gliomas, which can be further subdivided into astrocytomas, oligodendrogliomas, ependymomas, and glioastrocytomas [2,3]. Concerning the grade of malignancy, oligodendrocytomas and mixed gliomas are classified as II and III grade, while astrocytomas are subdivided into low-grade (LGA: pilocytic, grade I, and diffuse, grade II) and high-grade (HGA: anaplastic, grade III, and glioblastoma multiforme, GBM, grade IV) astrocytomas [4]. HGA are mostly treated by surgery, followed by radiation and chemotherapy [4]. However, because of the high infiltrating capacity of glioma cells, and despite great advancements of both radio-/chemo-therapeutic and surgical protocols, therapy of HGA still remains almost ineffective, with less than 10% of patients surviving for more than three years [5,6,7].

A further problem concerns early identification of a brain tumor: for a variable period of time after the onset of cancer, patients may suffer from only unspecific symptoms, such as seizures and headache [4].

A critical aspect of brain tumors is their contrasting relationship with the blood–brain barrier (BBB), both in tumor diagnosis and therapy: if, on the one hand, BBB hampers identification and treatment of cancer by forming a barrier, which often opposes to drug access into the brain [8,9], on the other hand, paradoxically, BBB becomes leaky because of cancer, thus causing vasogenic brain edema, the most frequent and serious clinical complication of GBM [10]. Moreover, brain cancer cells are able to actively induce neuronal [11] as well as glial [12] cell death, and neurodegeneration, associated with cytotoxic edema [10,13]. These latter events are probably linked with the ability of cancer cells to produce and release into their environment extracellular vesicles (EVs), which contain a collection of different factors, able to promote cancer progression by suppressing immune response, while stimulating cancer growth and invasion, angiogenesis, and metastatic spreading [14].

Both cytotoxic and vasogenic edemas seem to depend on altered expression and/or localization of water channels, belonging to the aquaporin family (AQPs). Here we summarize the possible roles played by aquaporins in physiological brain functions as well as in brain tumors, in relationship with both kinds of edema.

## 2. Brain Circulation and Aquaporins

The cell membrane is a highly integrated system, dynamically responsive and reactive to environmental conditions, and linked to many other cell structures, such as the cytoskeleton (inside the cell) and the extracellular matrix (outside) [15]. Importantly, membrane molecules can form domains of different sizes (from nano- to micro-sized) in reversible, non-uniform, non-random manner [16]. These domains, by interacting with other cell structures, cause partitioning of proteins and lipids, and generation of membrane corrals characterized by high densities of specific molecular complexes [15]. A fundamental property of cell membranes is their capacity to allow rapid and highly regulated water transport. Fluid transport in tissues actually follows two routes: (i) transcellular, between apical and basal cell membranes, driven by osmotic stimuli; and (ii) paracellular, in the inter-cellular spaces, across cell-to-cell junctions [17,18].

### 2.1. Brain Circulation

The brain parenchyma has been traditionally considered a lymphatic vasculature-lacking organ, from which the necessity to envisage alternative pathways for clearance of solutes and water from the interstitial fluid (ISF) of the extracellular space (ECS), and from the cerebrospinal fluid (CSF). CSF is produced by active secretion from the choroid plexus, and flows in the ventricles and the subarachnoid space (SAS). In the brain, four distinct water compartments can actually be recognized: intracellular fluid (ICF), ISF, CSF and blood. ICF composition shows significant differences when different cell types (i.e., neurons and glial cells) are compared; for example, neurons keep chloride concentration lower than glial cells, thanks to the presence in their membrane of potassium chloride cotransporter (KCC) 2 [19]. Moreover, astrocytes have a higher water permeability than neurons because of high concentration of the aquaporin protein AQP4 (see below) [20]. Interestingly, ISF and CSF have similar composition, thus suggesting a CSF-ISF dynamic exchange of solutes and water. Since about thirty years ago, many observations suggested that CSF could flow throughout the brain along paravascular spaces running along cortical arteries [21,22]. Based on these observations, as well as on the functional similarity with the peripheral lymphatic system, and on the important role played by glial cells in cerebral water flowing, other researchers suggested the existence, throughout the brain, of a water-exchanging network, driven by cerebral arterial pulsation, which was termed *glymphatic* system [20,23,24]. 

It has also been reported that CSF is finally drained into extracranial lymphatic vessels and lymph nodes (LNs). Recently, in a transgenic mouse model with complete aplasia of the dural lymphatic vessels, Aspelund et al. [25] observed complete abrogation of transport from the subarachnoid space into LNs. Based on this observation, the Authors suggested that CSF flux into the LNs is mediated through a dural lymphatic network. Similar observations have also been reported by Louveau et al. [26], and have also been vivaciously discussed in the context of removal from the brain of waste products, such as the amyloid Aβ-peptide and the tau protein [27].

Finally, as far as the vascular compartment is concerned, its almost complete independence from the other water compartments is well known and due to the blood–brain-barrier (BBB), a highly selective structure formed by brain capillary endothelial cells (BCECs) [28,29,30,31,32,33,34,35]. In most of our body, the endothelial cells that form the walls of capillaries have gaps, while BCECs are tightly sealed together thanks to the tight junctions (TJs). Moreover, BCECs do not possess aquaporins (see below) [36].

TJs are formed by many different proteins, among which claudins [37,38] and occludin [39,40], and the quality of the BBB function depends on correct synthesis, post-translational modification, and peripheral localization of these proteins. Importantly, formation and maturation of TJs depend in turn on the brain microenvironment and on glial cells and pericytes [41]. By using an in vitro model of BBB, we demonstrated that not only astrocytes but also neurons can affect BBB formation and maintenance [42,43,44]; these effects are probably due to secretion of angiogenic factors, such as vascular endothelial growth factor (VEGF) and fibroblast growth factor-2 (FGF-2), which are released, at least in part, through extracellular vesicles [45,46]. In the same co-culture system it was also found that BBB can be damaged by factors present in the serum of patients affected by multiple sclerosis, and that BBB breaking is accompanied by a decrease of the synthesis, and peripheral localization, of occludin [47]. 

### 2.2. General Properties of Aquaporins

Although partially due to passive co-transport with other molecules and ions [48], transcellular water flow is mostly mediated by specialized water channels called aquaporins (AQPs). Since their discovery in erythrocytes [49] and renal tubules [50,51], at least 13 different isoforms of AQPs have been identified as channels widely expressed in various fluid-transporting epithelial and endothelial cells in mammals, and able to modulate the capacity of cells to control their volume, in response to a changing osmotic environment [18,52,53]. Different AQP isoforms have different tissue localization and specific functions: AQP0, AQP1, AQP2, AQP4, and AQP5 are water channels, while AQP3, AQP7, AQP9, and AQP10, also called aquaglyceroporins, are also able to transport other polar molecules, such as glycerol and urea, and probably also some non-polar gases, such as CO_2_ and O_2_ [36]. In addition, a third group of AQPs, with low homology to the already known ones, has recently been identified and termed superaquaporin family [54]. Proteins of this latter group, also referred as unorthodox aquaporins, include AQP11 and AQP12, two AQPs present in the cytoplasm and probably involved in the maintenance of organelle volume [55], AQP6 and AQP8 [56].

AQPs exist in cell membranes as tetramers; each monomer is a 28–30 kDa protein, which contains six trans-membrane α-helices, lining an independent channel; the narrowest part of this pore contains conserved Asn-Pro-Ala (NPA) motifs. Assembly of the four subunits also determines formation of a central space, which has been suggested to be permeable for gases, in AQP1, AQP4 and AQP5 [57,58,59,60]. 

Although AQPs generally form homo-tetramers, AQP4 can also form hetero-tetramers. Two different isoforms of AQP4 monomers exist [61,62,63]: (i) a longer AQP-M1 isoform, with translation initiation at met 1; and (ii) a shorter AQP-M23 isoform, with translation initiation at met 23. The two isoforms can form both homo- and hetero-tetramers with different localization and properties; of particular interest (see below) is the ability of the M23-containing tetramers to assemble into supramolecular complexes, known as *orthogonal arrays of particles* (OAPs) [36,64,65,66].

### 2.3. Aquaporins (AQPs) Expression and Function in the Nervous System

Many AQPs, (AQP1, AQP3, AQP4, AQP5, AQP6, AQP8, AQP9 and AQP11) have so far been identified in the CNS, the most represented of which are AQP1, AQP4 and AQP9 [18,67,68,69]. AQP1 is expressed in the choroid plexus epithelium and seems to be involved in the formation of CSF [70], while AQP4 is present both in astrocytes [18,71] and neurons [72]. No AQP is present in the BCECs, which constitute the anatomical basis of the BBB. However, AQP4 distribution in astrocytes is highly polarized to astrocytic endfeet which contact the blood vessels at the BBB, as well as at the CNS-CSF interface (glia limitans) [73]. At these sites, AQP4 is included in the already mentioned OAPs, the formation and polarity of which are established during development, in parallel with maturation of the BBB [64,74]. The density of AQP4/OAPs is particularly high (100–400/μm^2^) at the sites where the astrocyte membrane directly contacts the perivascular as well as the superficial basal lamina, at the surface of the brain, while, when the membrane turns back from the basal lamina, the density of OAPs dramatically decreases to about 10–20/μm^2^ [75,76,77,78]. This peculiar AQP4 localization in the surroundings of cerebral capillaries suggests a role for AQP4 in extracellular fluid clearance. OAP formation and localization in astrocytes depend on their interaction with both intracellular scaffolding proteins, such as α-syntrophin [79], and extracellular proteoglycans, such as agrin, a heparan sulfate proteoglycan. Agrin in turn binds to α-dystroglycan, a component of the dystrophyn-dystroglycan complex, found at many sites in the CNS [76]. Interestingly, these complexes also contain the inwardly rectifying potassium channel Kir4.1, a protein involved in spatial buffering of K^+^ ions released, because of synaptic activity, into the extracellular space. K^+^ ions are taken up by astrocytes and water osmotically follows them through the AQP4 pores [41]. In general, polarized AQP4 expression characterizes the boundaries between the brain and various fluid compartments, thus suggesting its involvement in regulation of water flow in and out of the brain [36].

AQP9 is a member of the aquaglyceroporin family, probably involved not only in water flux across the plasma membrane, but also in permeation of monocarboxylates, such as lactate and β-hydroxybutyrate, and other solutes, such as glycerol, purines, pyrimidines and urea [80]. AQP9 expression has been evidenced in many sites, and different cytotypes, including astrocytes and catecholaminergic neurons [80,81,82,83].

In addition to the main AQPs, at a lower concentration and with still unknown physiologic functions, other AQPs have been reported to be present in the CNS, based on reverse transcriptase-polymerase chain reaction (RT-PCR) [84], immunohistochemistry, and immunofluorescence [85]. 

AQP8, an AQP widely expressed in the reproductive systems [86], in the digestive system [87], and kidney [88], has been, for example, also evidenced in various CNS areas, such as piriform cortex, choroid plexus, and hippocampus. Several studies showed that AQP8 channels are present in the inner mitochondrial membrane of various tissues, suggesting a role in the osmotic equilibrium of these intracellular organelles and perhaps in the maintenance of acid–base equilibrium [85]. 

AQP3 and AQP5 seem to also be present in the CNS, with a distribution patterns similar to AQP8 [85]. Moreover, AQP6 has been reported to be expressed in cerebellum [89] and retina [90].

Finally, expression of the superaquaporin AQP11 in the CNS, and in particular in Purkinje cells of the cerebellum [91], in the epithelium of the choroid plexus and perhaps in BCECs [54] has been reported. Its function in these sites, however, remains to be clarified.

## 3. Brain Tumors and Aquaporins

### 3.1. Epidemiology of Brain Tumors

Primary CNS cancers are among the top 10 causes of tumor-related deaths in the United States, accounting for approximately 1.4% of all cancers and 2.4% of all cancer-related deaths [92]. In European countries, standardized incidence of primary brain tumors comprises between 4.5 and 11.2 cases/100,000 men, and between 1.6 and 8.5/100,000 women [1]. Increases in the incidence of malignant brain tumors have been attributed to several factors: improved diagnostic procedures, such as computed tomography and magnetic resonance imaging (MRI); a greater availability of neurosurgeons and changing patterns of access to medical care. Although analytic epidemiologic studies have suggested involvement of environmental factors, no specific risk factor, accounting for large percentages of brain tumors, has been identified up to now [92].

As mentioned, most primary tumors in the adult are gliomas (42% of all primary CNS tumors and 77% of all malignant ones). Among gliomas, diverse histological lineages can be distinguished, including astrocytoma, oligodendroglioma, and mixed oligoastrocytoma. For all these cytotypes, both low-grade and high-grade variants are known, and all have the potential to evolve into highly malignant tumors, resistant to treatment, and named glioblastoma multiforme (GBM or World Health Organization Grade IV astrocytoma), the most common and aggressive primary brain cancer in adults [93].

The average age at which gliomas first arise is different for the different histological lineages. For example, anaplastic astrocytomas (AAs; grade III) develop at approximately 40 years, while GBM has a peak of incidence between 65 and 74 years of age. 

Malignant astrocytomas are typically invasive and highly infiltrating, and effective resection is therefore unlikely. As a consequence, patients with CNS tumors have a poor prognosis: according to the data collected by the Surveillance Epidemiology and End Results Program, the median survival time is approximately 3.5 months for patients >65 years old at diagnosis, and about 10 months for those <65 years [94]. The survival time is also influenced by additional factors, among which the histological features of the neoplasm, and the neurological condition and/or the general physiological status of the patient. In general terms, the effect of treatment on the overall prognosis of brain cancers is frustratingly low [93]. In a multicenter long-term study, patients treated with gliadel (bis-chloroethylnitrosourea, or carmustine) showed a median survival of 13.8 months versus 11.6 months of placebo-treated patients [95]. A median survival of 14.6 months was instead reported for GBM patients who underwent surgical resection, radiotherapy, and chemotherapy with temozolomide [96,97].

As already mentioned, management of GBM typically consists of surgery followed by radiotherapy or chemotherapy, or both. However, because of the high infiltrating capacity of glioma cells, all therapies remain almost ineffective, residual tumor seems to be inevitable and patients eventually succumb to disease [5,6,7,97].

### 3.2. Peritumoral Edema

Anaplastic astrocytomas (WHO grade III), and GBM (WHO grade IV) often arise in the cerebral hemispheres. Both species can derive from a low-grade astrocytoma (WHO grade II), but can also be diagnosed de novo, without previous signs of a precursor tumor [3].

**Figure 1:**
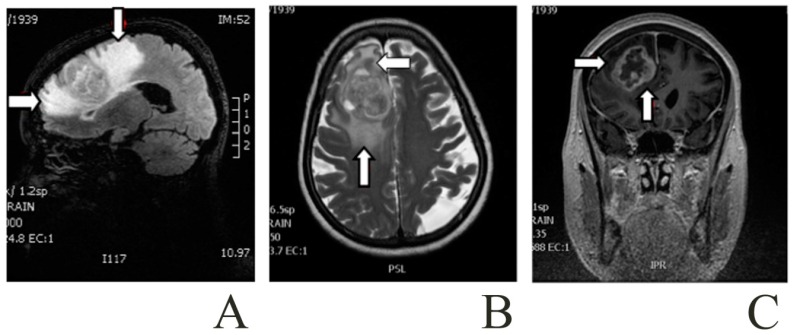
Patient with a right frontal glioblastoma and a marked area of peritumoral edema. Images from a Brain Magnetic Resonance. The arrows point to the peritumoral edema close to the glioma: (**A**) Sagittal T1-weighted; (**B**) Axial T2-weighted; and (**C**) Coronal T1-weighted contrast-enhanced with gadolinium.

Patients with malignant astrocytomas suffer from relatively uniform symptoms and signs, including increased intracranial pressure, which depends on the growing tumor mass, and the peritumoral cerebral edema [97] (Figure 1). Brain edema is indeed typically present in human brain cancers and affects both the course and outcome of pathology [98]. The appearance and effects of edema in clinical progression of brain cancers has been known since long ago in clinical practice, and has been extensively discussed in the scientific literature. Since the nineties, two main subtypes of brain edema (cytotoxic and vasogenic) were recognized, based on their pathophysiology [1,99,100]. In cytotoxic edema, cells swell because of malfunction of Na^+^/K^+^-ATPase and sodium retention, accompanied by water excess in the ICF; the blood–brain barrier (BBB) is meanwhile intact. On the other hand, the hallmark of vasogenic edema is breakdown of BBB, and the consequent loss of homeostasis in the neural parenchyma microenvironment. The two forms of edema, however, may share some basic mechanisms. Growing primary or secondary tumors are indeed able to release factors which promote proliferation of BCECs: thus, on the one hand BCECs loose their preformed TJs, and, on the other hand, the nascent microvessels do not have mature TJs; this brain–tumor interface does not constitute a competent barrier, and allows leakage of plasma ultrafiltrate into the parenchyma ECF. As explained above, under normal conditions, healthy BBB restricts the movement of molecules across the vascular endothelium, and water circulation into the brain is primarily driven by hydrostatic forces (cerebral perfusion pressure) and water co-transport with solutes and ions. When TJs, which normally link together BCECs, are disrupted, extravasation of intravascular solutes (and water) is allowed, and the normal homeostasis is lost: water flows into the parenchyma along hydrostatic gradients, with no opposing osmotic forces, thus causing fluid accumulation in the ECF, in the absence of cell volume modification [100].

The most widely studied permeability and angiogenic agent secreted by tumor cells is vascular endothelial growth factor (VEGF), which induces capillary permeability, endothelial proliferation, migration, and organization of new capillaries that lack tight junctions. All the CNS tumors normally associated with edema (i.e., glioblastomas, meningiomas, and metastases) have been reported to produce high levels of VEGF [97].

The mechanisms through which water is cleared from the ECF are less clear. By using radioactive and fluorescently labeled tracers, in animal models, it was found that, when intracranial pressure (ICP) increases, the CSF clearance of the labeled tracers decreases, suggesting activation of an alternative water clearance pathway [101]. Based on observations of this kind, several authors have hypothesized that the parenchymal route of extracellular fluid resorption, which becomes especially effective under high ICP and abnormal CSF flow dynamic, involves AQPs [100].

### 3.3. AQPs and Gliomas

As discussed above, a large part of fluid exchanges in the brain are mediated by specialized water channels called aquaporins, and are driven by osmotic and hydrostatic pressure gradients [41].

Several studies have evidenced an involvement of aquaporins in multiple aspects of malignant brain tumor pathogenesis, such as promotion of tumor cell motility and invasiveness, as well as edema formation, and improvement of glycolytic tumor cell metabolism, under hypoxic conditions. [102]. Glioma cells are extremely invasive: dynamically changing their own volume, they navigate throughout the normal parenchyma along the tortuous and narrow extracellular spaces by following a water flux largely generated by AQPs [103,104]. On the other hand, edema represents for the patients a further critical aspect of the pathology. Given the central role of aquaporins in brain cancer growth and invasiveness, as well as in edema formation, AQPs can also represent a potential target for brain cancer therapy [100].

#### 3.3.1. AQP1

**Figure 2:**
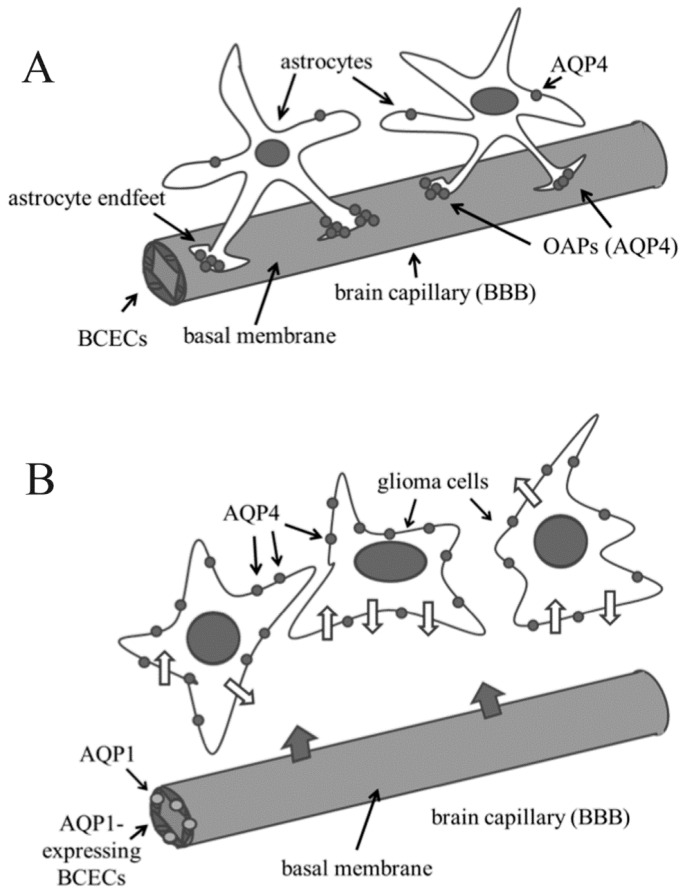
Alteration of expression and/or localization of aquaporin 1 (AQP1) and AQP4 in brain cancer cells respect to normal glial cells. (**A**) In normal brain, brain capillary endothelial cells (BCECs) do not express AQP1, while AQP4 is mainly present in the astrocyte endfeet, where it forms orthogonal arrays of particles (OAPs); (**B**) In gliomas, BCECs express AQP1, thus probably allowing an increase of blood–brain-barrier (BBB) permeability (large grey arrows indicate water flux from blood to brain), while AQP4 is delocalized: endfeet (and OAPs) are no more visible and AQP4 is found throughout the plasma membrane of glioma cells, thus probably increasing water trafficking across the cell membranes (narrow white arrows on glioma cell membrane).

By differential gene expression analysis [105], and immunohistochemistry [106], as well as by reverse transcriptase polymerase chain reaction, complementary DNA gene array, and Western blot analysis [107], up-regulation of AQP1 was clearly demonstrated in high grade-astrocytomas. Moreover, AQP1 expression has been reported to be somehow proportional to the grade of malignancy [106,107,108]. Interestingly, AQP1 expression in brain cancer is associated with BCECs, which do not express this AQP in normal brain; this observation suggested that AQP1 up-regulation can be involved in vasogenic edema [70,109,110] (Figure 2).

The signals that induce AQP1 expression in the endothelium of brain tumors are, however, still not completely understood, but might include production and release from cancer cells VEGF, which can in turn increase vascular permeability by stimulating BCEC proliferation [100]. Actually, production of VEGF is regulated, at the transcriptional level, by hypoxia through activation of the transcription factor known as hypoxia induced factor (HIF) [111]. Moreover, since the first description done by Otto Warburg in 1931 [112], the effect of hypoxic conditions on cancer cells has been well recognized: in malignant cells (and in GBM in particular) indeed, hypoxia stimulates anaerobic glycolysis (Warburg effect), with an increase of glucose consumption and lactate production [113,114,115], which can persist even under normoxic conditions [102]. It has been suggested that increase of lactate induces ICF acidification and promotes excess H^+^ extrusion into the ECF (probably together with water), thus providing an important function for AQP1 upregulation in cancer cells [70]. In general terms, it was found that AQP1 expression, as well as the expression of other proteins, among which lactic dehydrogenase (LDH) and the protease cathepsin B (which facilitates degradation of the extracellular matrix), correlates with glycolytic levels [70]. Taken together, these modifications should allow improvement of glioma cell migration and invasion [102]. The importance of AQP1 in determining malignancy of cancer cells is also confirmed by the observation that some AQP1 polymorphisms can be used as survival prognosis factors in patients with GBM [116].

Interestingly, it has been recently reported that AQP1 (and AQP4) are upregulated even in benign subependymomas [117]. Moreover, AQP1 was also detected in meningioma cells and capillaries, which invade the dura [118], as well as in association with the Na-K-2Cl cotransporter (NKCC1), an ion transporter that can affect fluid movements in different kinds of brain lesions [119,120]. 

In conclusion, many studies have reported the importance of AQP1 in cerebral edema, tumor growth, angiogenesis, and neoplastic invasiveness; one of the possible mechanisms involved seems to be the induction of endothelial cell migration, depending on a water influx into the cells, with consequent expansion of their protrusions (lamellipodia) [102,103,121,122]. Based on these observations, many Authors suggested that AQP1 blockers might function as potent anti-brain tumor edema agents. Interestingly, in the promoter of AQP1 gene, steroid responsive elements are present [123], which could be responsible for regulation of AQP expression and, in turn, for the anti-brain tumor edema action of glucocorticoids [106,124,125].

#### 3.3.2. AQP4

AQP4 is the main water channel in the brain and is expressed throughout several CNS structures, such as the ependymal cell layer lining the lateral ventricle and aqueduct, the pia mater, the choroid plexus, the hypothalamus, and the cerebellar Purkinje cells [67,68,69,126].

As discussed above, AQP4 is primarily an astroglial membrane protein, localized to the astrocytic endfeet which point to the cerebral microvessels [100]; in this location, AQP4 molecules contribute to form OAPs, acting as a key functional component of BBB (Figure 2).

Because of this peculiar location, AQP4 has been suggested to be involved in different aspects of brain edema pathogenesis, probably with different effects depending on the nature of the disease (for example, cancer or traumatic damage). However, the meaning of AQP4 upregulation has not yet been fully understood: (i) acting as a cause, due to a primary and abnormal tissue response; or (ii) being only a side effect of a tissue response, aimed at eliminating fluid excess resulting from capillary leakage [76,77,78]. Independent of the real role of AQP4, there is clear evidence of a relationship between edema formation and AQP4 upregulation. For example, it has been reported that, after an ischemic stroke, AQP4 accelerates edema development [127,128], and AQP4 clearly increases after traumatic brain injury [9,129,130]. Vasogenic edema has a great effect on the morbidity and mortality associated with malignant brain tumors, and AQP4 has a major role in its pathogenesis [102]. Bloch and Malley tested the role of AQP4 in extracellular edema, measuring intracranial pressure (ICP) and brain water content in both wild-type and AQP4-deficient mice, after a continuous infusion of artificial CSF into the brain extracellular space. They found that AQP4-deficient mice had significantly higher brain water content and two-fold greater ICP than wild-type animals, suggesting a delayed fluid clearance in these AQP4-deficient mice [100]. 

Interestingly, a correlation has been reported between AQP4 expression and the incidence of epileptic seizures in GBM patients: in particular, it was found that, even if all GBM patients enrolled expressed similar amounts of AQP4 mRNA, patients with seizures had a higher amount of AQP4 in the cell membranes, thus suggesting a post-transcriptional regulation of AQP expression [131].

An important field of investigation concerns other molecules that could interact with AQP4 in edema formation. As in the case of AQP1 upregulation in brain cancer, one of these molecules is VEGF, which can promote tumor neovascularization, vessel permeability, and extravasation of plasma proteins into extracellular brain spaces. All these effects cause *per se* extensive damage of BBB allowing plasmatic macromolecules to enter the interstitial space, where they produce an obvious change in osmotic pressure. It has been reported that AQP4 is positively regulated by VEGF [128,132,133]. In this context, VEGF should be the real inducer of vasogenic edema, while upregulation and intracellular redistribution of AQP4 should represent a protective reaction, aimed at avoiding a secondary cytotoxic brain edema, by facilitating reabsorption of excess fluid [134]. 

In general terms, AQP4 is clearly upregulated in brain tumors, including pilocytic astrocytoma and glioblastoma [41,76,77,78,106,124,125,135]. A direct correlation was also found between AQP4 +expression and patients’ mean survival time. AQP4 expression was high in WHO I pilocytic astrocytomas (perhaps because of their peculiar vessel morphology, with a microvascular proliferation and for the presence of glomeruloid bodies), in WHO III anaplastic astrocytomas, and in WHO IV glioblastomas (the most aggressive forms of primary brain tumors), whereas WHO II tumors showed significantly lower AQP4 levels. Intracellular AQP4 redistribution was also significantly lower in tumor infiltration areas than in the tumor center, suggesting that the alteration of AQP4 expression pattern is specific for neoplastic regions [76,77,78].

A variety of reports suggest that AQP4 is involved in promoting cancer cell migration, and, indeed, AQP4 deletion markedly impaired astrocytoma cell migration and invasion [67,68,69,106,124,125,136]. However, which is the underlying molecular mechanism of this effect? One possibility is that the water flux mediated by AQP4 facilitates rapid modification of cell volume and shape, thus improving movement [137]. In particular, AQP4 might allow water flow across the plasma membrane at the level of the leading cell protrusions (lamellipodia), with an effect on their polarization, total number and size. At same time, these structures undergo rapid actin cytoskeleton rearrangements and changes in osmolality of the cortical layer of cytoplasm [67,68,69]. Moreover, it has been suggested that AQP4-mediated water permeability at these sites, and tumor invasion, could be down-regulated by PKC activation, and AQP4 phosphorylation [104].

Interestingly, AQP4 expression was found to be higher in the peritumoral area than in the tumor core, thus suggesting a correlation between the highest AQP4 expression and the highest ability to invade the surrounding tissue [128]. 

Probably, the most striking aspect of glioma cells respect to normal astrocytes is the completely changed organization of AQP4 complexes: as mentioned above, in the normal brain AQP4 forms OAPs specifically localized to the astrocytic endfeet; in glioma cells typical endfeet are no more recognized, and AQP4 is not only up-regulated but also redistributed along the entire cell membrane [138] (Figure 2), with a parallel loss of the normal astrocyte-BCEC contacts at the BBB [139]. As the extracellular matrix (ECM) plays an important role in the polarized localization of OAPs, AQP4 delocalization probably in turn depends on modification of ECM: it has indeed reported that, besides AQP4, dystroglycan, agrin and the matrix metalloproteinases (MMP) 2, 3 and 9 also undergo altered expression in human primary glioblastomas [140]. In particular, an agrin loss has been described, probably due to its degradation by MMP 3 [41]. 

#### 3.3.3. AQP8

The physiopathologic functions of AQP8 in brain tumors are largely unknown. Its location is, however, different from that of AQP1 and AQP9, suggesting a specific role for this AQP. In a series of 75 astrocytomas of different grades, Zhu et al. [141] showed an intracellular upregulation of AQP 8 in both low- and high-grade astrocytomas, compared with normal brain tissue, with a direct correlation to the cancer grade.

#### 3.3.4. AQP9

AQP9 was suggested to play a role in post-ischemic edema [142], and, given its permeability to monocarboxylates, in the clearance of lactate from the ischemic focus [136]. Changes in AQP9 expression may be the result of glial cell attempt to react to hypoxic and ischemic conditions, by facilitating clearance of lactate and glycerol, respectively. Thus, AQP9 could play a role in normal cell metabolism, under physiologic conditions, and also increase cell stress tolerance, under hypoxia and other pathological conditions [102]. Increased AQP9 expression, both at the level of mRNA and protein, has been also observed in astrocytic tumors of all grades [76,77,78,143]. Jelen et al. [80] reported, however, that in glioblastoma biopsies, unlike AQP4, AQP9 is expressed only in a subset of malignant astrocytic cells and in leukocytes, which infiltrate the tumor.

## 4. Novel Routes for Aquaporin Trafficking

Like other cancer cells, brain tumor cells produce and release into their environment extracellular vesicles (EVs), membrane structures that can derive either directly from the plasma membrane (ectosomes), through a process resembling virus budding, or from an intracellular organelle called multivesicular body (exosomes) [14,144]. EVs contain different species of mRNAs, proteins and microRNAs, which, once received by surrounding cells can alter their phenotype. EVs are also used by tumor cells to escape immune surveillance, and to discard proteins that could otherwise be able to counteract continuous proliferation and transformation of EV-producing tumor cells [14,145]. 

Although the ability of brain cancers to produce EVs has been clearly demonstrated, it is not known whether EVs are also used for AQP trafficking and redistribution. Interestingly, however, in other tissue systems, the presence of AQPs in EVs has been reported: for example, AQP2, the vasopressin-dependent AQP expressed in kidney, has been found to be secreted into urine as exosomes [146,147,148], and similarly a fraction of AQP1 is released into exosomes from maturing reticulocytes [149], as well as from kidney [150]. It is therefore likely that EVs are also used by brain cancer cells to redistribute AQP channels, in order to allow faster movement of fluids that contain molecules of metabolic importance. In addition, the presence in biological fluids of EVs carrying different classes of AQPs should be perhaps of help for early diagnosis of brain tumors, and/or of their progression.

## 5. Concluding Remarks

AQPs seem to play a variety of important roles in the normal brain, by coordinating water (and solutes) trafficking among the different fluid compartments of the nervous system. Exchange of water between the blood, CSF, ECF and ICF is obviously a dynamic process, but it can undergo significant modifications, both in volumes and direction, under pathological conditions, including brain cancers, as demonstrated by formation of different kinds of edema, besides the main pathology.

In the case of brain cancer, specific up-regulation of some AQPs, as well as their involvement in brain edema formation, has been consistently reported by many Authors. Moreover, recent observations have also suggested that the intensity of edema could correlate with specific AQP polymorphisms, as shown in a study on the AQP5 promoter A(-1364)C polymorphism, which is positively correlated with the intensity of brain edema in meningioma patients [151]. Based on all these findings, it has been suggested that AQPs could represent an important target in cancer/edema treatment. However, attempts to find molecules able to efficiently inhibit AQP channels have not quite been successful thus far, due to the following two main points: (i) the high number of AQP molecules in the cell membranes, together with the spatial restrictions of these structures [152]; and (ii) the necessity to facilitate drug delivery across the BBB [8]. 

Actually, it has been reported that an anti-AQP4 specific monoclonal antibody (aquaporumab) can compete and efficiently counteract binding to AQP4 of pathogenic autoantibodies produced in neuromyelitis optica (NMO), an inflammatory disease affecting the optic nerve and spinal cord [36]. Molecules of this kind might be also useful in reducing AQP4 activity in malignant gliomas [122]. A second kind of strategy might include use of small RNAs, complementary to specific regions of AQP4 mRNA (siRNAs): it has been reported, in fact, that down-regulation of AQP4 using this approach can induce cancer cell apoptosis [60,69].

In conclusion, given the importance of AQPs not only in cancer but also in many other neurologic pathologic conditions, such as traumatic injury, and stroke, it will be critical to improve the research in this field, aiming at finding out new drugs able to cross the BBB and to limit AQP up-regulation.
